# Automated segmentation and classification of supraspinatus fatty infiltration in shoulder magnetic resonance image using a convolutional neural network

**DOI:** 10.3389/fmed.2024.1416169

**Published:** 2024-09-03

**Authors:** Juan Pablo Saavedra, Guillermo Droppelmann, Carlos Jorquera, Felipe Feijoo

**Affiliations:** ^1^School of Industrial Engineering, Pontificia Universidad Católica de Valparaíso, Valparaíso, Chile; ^2^Clínica MEDS, Santiago, Chile; ^3^Harvard T.H. Chan School of Public Health, Boston, MA, United States; ^4^Facultad de Ciencias, Escuela de Nutrición y Dietética, Universidad Mayor, Santiago, Chile

**Keywords:** classification, deep learning, fatty infiltration, MRI, supraspinatus

## Abstract

**Background:**

Goutallier’s fatty infiltration of the supraspinatus muscle is a critical condition in degenerative shoulder disorders. Deep learning research primarily uses manual segmentation and labeling to detect this condition. Employing unsupervised training with a hybrid framework of segmentation and classification could offer an efficient solution.

**Aim:**

To develop and assess a two-step deep learning model for detecting the region of interest and categorizing the magnetic resonance image (MRI) supraspinatus muscle fatty infiltration according to Goutallier’s scale.

**Materials and methods:**

A retrospective study was performed from January 1, 2019 to September 20, 2020, using 900 MRI T2-weighted images with supraspinatus muscle fatty infiltration diagnoses. A model with two sequential neural networks was implemented and trained. The first sub-model automatically detects the region of interest using a U-Net model. The second sub-model performs a binary classification using the VGG-19 architecture. The model’s performance was computed as the average of five-fold cross-validation processes. Loss, accuracy, Dice coefficient (CI. 95%), AU-ROC, sensitivity, and specificity (CI. 95%) were reported.

**Results:**

Six hundred and six shoulders MRIs were analyzed. The Goutallier distribution was presented as follows: 0 (66.50%); 1 (18.81%); 2 (8.42%); 3 (3.96%); 4 (2.31%). Segmentation results demonstrate high levels of accuracy (0.9977 ± 0.0002) and Dice score (0.9441 ± 0.0031), while the classification model also results in high levels of accuracy (0.9731 ± 0.0230); sensitivity (0.9000 ± 0.0980); specificity (0.9788 ± 0.0257); and AUROC (0.9903 ± 0.0092).

**Conclusion:**

The two-step training method proposed using a deep learning model demonstrated strong performance in segmentation and classification tasks.

## Introduction

Rotator cuff tears (RCTs) are a prevalent musculoskeletal shoulder condition that affects millions of people worldwide, regardless of sex (1, 2). This degenerative and progressive condition becomes increasingly common with age in the general population (3), leading to significant economic consequences for patients and healthcare systems alike (4, 5). The magnitude of tear size, muscle atrophy, and fatty infiltration are important variables in predicting the prognosis of patients (6, 7). Specifically, low levels of fatty infiltration have been shown to have significantly better outcomes than those with more severe conditions, as they are less likely to experience re-tears (7, 8). Therefore, identifying specific stages of fatty infiltration and the supraspinatus muscle is crucial in accurately predicting patients’ prognoses, particularly for those that are to be exposed to a major surgery or in population of high risk with such as older patients. For this purpose, magnetic resonance image (MRI) is one of the most commonly used medical imaging techniques available for the detection of RCT and fatty infiltration, owing to its high diagnostic accuracy (9). However, patient access to MRI results may take several days due to the large number of exams and the time specialists can dedicate to this task. Therefore, developing tools that can speed-up this process, while having a high accuracy in identifying fatty infiltration, can help reduce waiting times suffered by patients and the burden faced by medical experts.

Goutallier et al. (10) proposed one of the most widely used qualitative scales for identifying supraspinatus fatty infiltration, consisting of five stages ranging from 0 (normal muscle) to 4 (severe fat accumulation). Although Goutallier’s scale was originally developed based on CT scan analysis, it has been adapted for use with MRI. Fuchs et al. (11) proposed a new scale by combining the previously defined stages in Goutallier’s work. Specifically, levels zero and one were merged to create the normal stage, level two was redefined as moderate, and level three or four were considered to represent severe fatty infiltration. However, there has been some controversy over the adaptation of the original scale for use with MRI (12). Furthermore, reducing inter-observer variability when assessing rotator cuff quality from MRI remains a major challenge in diagnostic imaging (13).

On the other hand, deep learning algorithms, especially convolutional neural networks (CNNs), have rapidly become the preferred methodology for analyzing medical images (14–16). Some of the most commonly used deep learning architectures for computer vision tasks include Inception-v3, ResNet50, VGG19, and U-Net (17–20). However, due to complexity of medical image datasets and smaller size compared to other sources of data, transfer learning has become a suitable approach for building and training deep learning models in clinical research. With transfer learning, most of the proposed models for medical diagnosis are based on pre-trained models from the ImageNet dataset and trained using transfer learning techniques (21). This technique involves using a well-trained model from a non-medical source dataset, such as ImageNet, and re-training it in a target dataset, such as medical images, including MRIs (22–24).

Most of the existing deep learning applications are based on supervised training, a commonly used technique for classification using medical images. However, supervised training requires labeled images for the models to learn from their structure. Additionally, in supervised learning, in order to improve the model’s performance, researchers manually select the region of interest (manual segmentation). However, manual segmentation is a time-consuming task, and manual labelling from medical experts is not always available (25). Therefore, to address these limitations, unsupervised training for segmenting the region of interest could be a viable solution. In the context of identifying shoulder fatty infiltration, four recent and highly important articles addressing this problem or closely related have been published. Three of these studies focused on magnetic resonance images (22, 23, 26) while only one utilized CT scans (27). However, all these studies relied on annotated data, which means that each image was manually labeled by an expert to create an image and corresponding infiltration level pairs, or each image was manually segmented to generate a corresponding segmentation mask for that specific image.

In order to address the gap in the literature, the objective of this research is to develop and assess a two-step deep learning framework. The first step performs and automated detection the region of interest (segmentation of the region of interest), while the second step uses the information from the segmentation model to classify the region of interest into one of the Goutallier’s fatty infiltration levels using MRI images, hence, fully automating the process of identifying the Goutallier’s fatty infiltration levels via the usage of deep learning techniques (segmentation and classification hybrid framework).

## Materials and methods

### Study design

This research was designed as a retrospective, single-site study, following the guidelines outlined in the Strengthening the Reporting of Observational Studies in Epidemiology (STROBE). Patient records were exclusively obtained from MRI examinations conducted at the MEDS Clinic in Santiago, Región Metropolitana, Chile. The study started on September 25th, 2020.

### Learning approach

**Figure 1:**
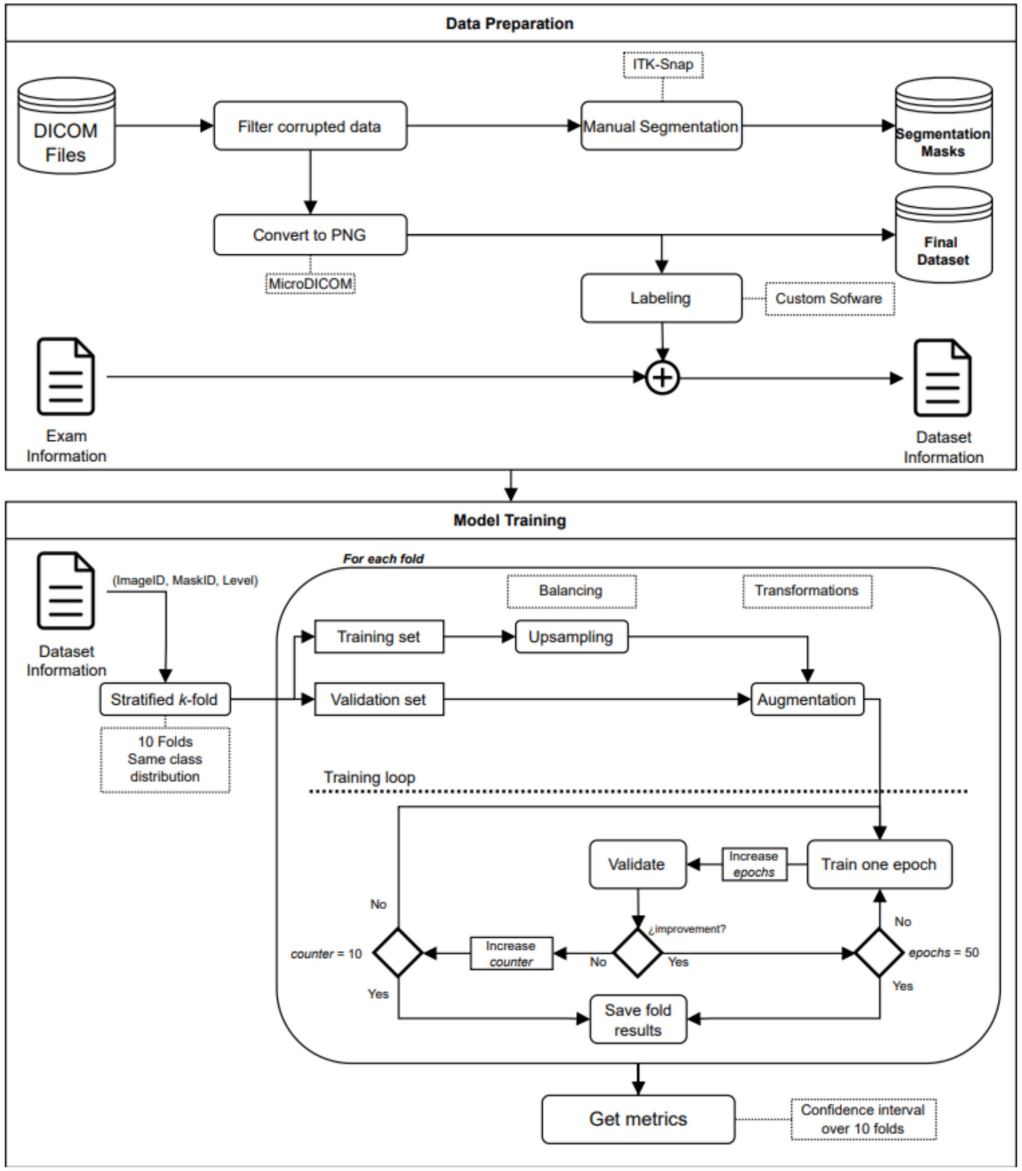
Workflow diagram.

**Figure 2:**
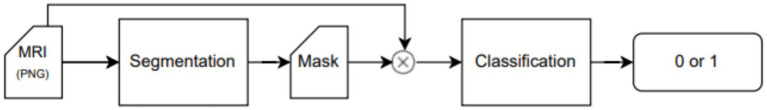
Diagram of the end-to-end model sequence.

An end-to-end deep learning model was developed to classify the patient risk based on the fatty infiltration of the supraspinatus muscle. The training process was performed in a two-step fashion. In the first step, we trained a segmentation model to extract the region of interest from the image. In the second step, we trained a classification model to determine if there was a risk or not for further surgery based on the level of fatty infiltration in the region of interest detected in the first step. Both models (segmentation and classification) are trained independently and non-recursively. However, segmented images from the first step (segmentation model) are used to train the classification model. Therefore, the training process of the classification model, as well as the testing phase, are performed using results from the segmentation model (segmented images). The training process and workflow of the proposed two-step model is described in Figure 1 as well as in Figure 2.

#### Dataset characteristics

The medical institution provided all the data, consisting of 900 DICOM files corresponding to unique exams. Each file corresponds to a T2-weighted Y-view MR sequence of the shoulder. Furthermore, we extracted all 900 medical reports associated to each of the DICOM files. The medical reports were, authored by three different radiologists. These reports used various scales or standards to document the fatty infiltration or degeneration stage. To ensure accurate labeling, we enlisted the expertise of an experienced radiologist who manually labeled the dataset. Moreover, images with diagnostic uncertainties underwent manual segmentation under the supervision of another radiologist, ensuring detailed and reliable annotations.

**Figure 3:**
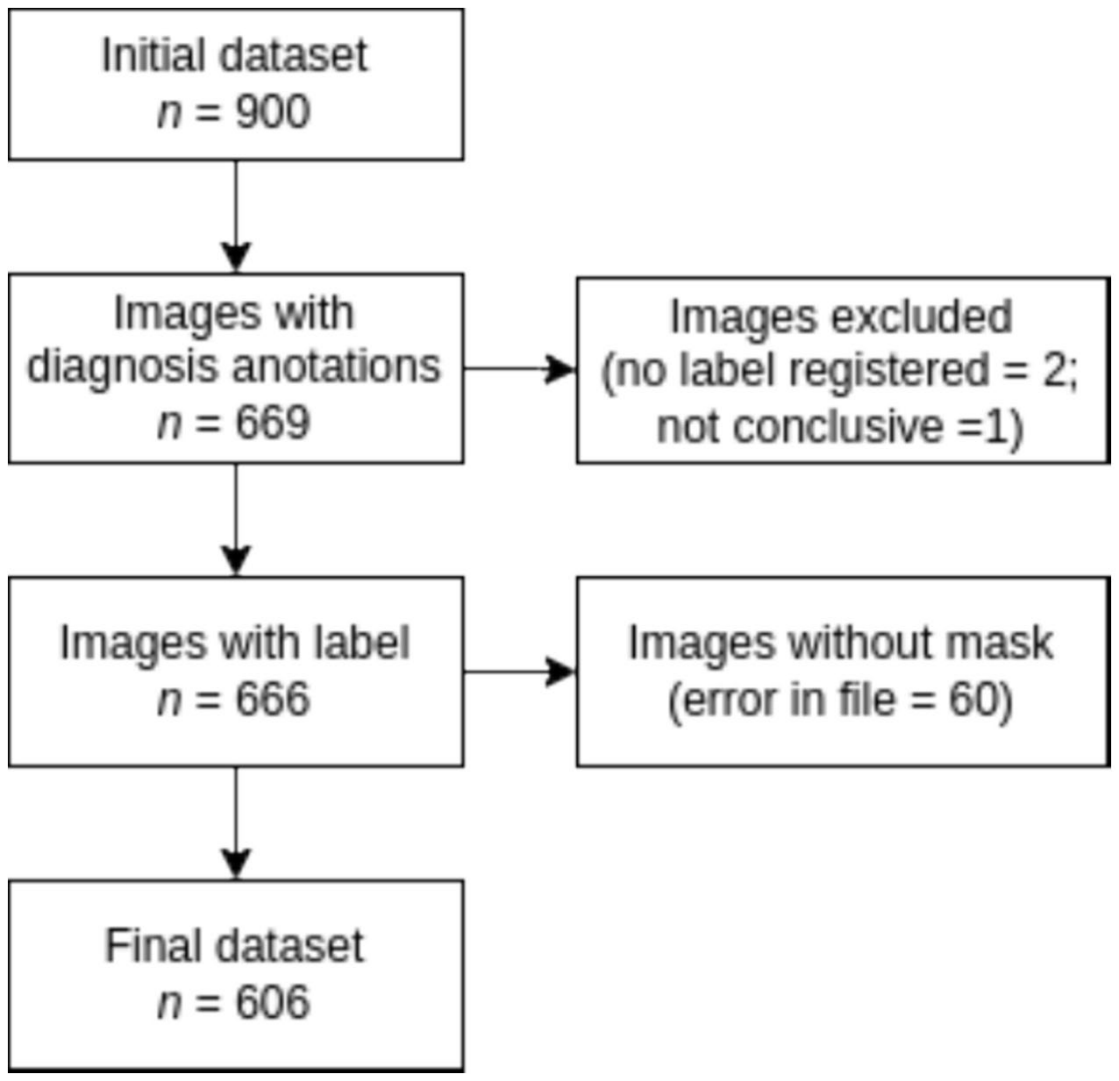
Flowchart for dataset selection.

According to Figure 3, the labeling process resulted in 666 registered images, with one being marked as inconclusive and two remaining unregistered. Additionally, there were 60 images for which segmentation masks could not be created due to a file error. Consequently, our ground truth dataset comprises 606 labeled images along with their corresponding segmentation masks. Table 1 provides an overview of the image label counts, indicating 403, 114, 51, 24, and 14 for Goutallier 0, 1, 2, 3, and 4, respectively. More than 82% of the images fall into grades 0 or 1, indicating a significant imbalance towards lower fatty infiltration grades. The female group exhibited a greater number of samples in the higher grades compared to the male group. Furthermore, except for the observed mean age in the Goutallier 0 group (*p* < 0.05), there were no significant differences between the female and male groups across Goutallier levels in terms of proportions or mean age.

**Table 1:** Patient data distribution Goutallier’s level by sex.

Goutallier level	*N* (%)	Female		Male		*p*-value	
		*N* (%)	Age mean (SD)	*N* (%)	Age mean (SD)	*N*	Age
0	403 (66.50)	140 (35)	53.06 (10.55)	263 (65)	49.24 (13.13)	0.477	***
1	114 (18.81)	74 (65)	61.50 (10.37)	40 (35)	63.58 (8.17)	0.465	0.371
2	51 (8.42)	31 (61)	66.65 (9.53)	20 (39)	66.40 (10.13)	0.447	0.992
3	24 (3.96)	16 (67)	68.88 (7.74)	8 (33)	64.25 (7.59)	0.424	0.230
4	14 (2.31)	13 (93)	67.31 (7.33)	1 (7)	N.A.	0.354	0.8
Total	606 (100)	274 (45)	58.47 (11.67)	332 (55)	52.42 (13.81)	0.483	

Mann–Whitney or *t*-test were used to compute the significance (alpha 0.05).

#### Dataset preparation

The DICOM file format is extensively adopted as a standard for medical images in clinical settings. A DICOM data object consists of multiple attributes, including fields such as name, ID, and more. It also incorporates a distinct attribute that contains the image pixel data. In order to enhance the efficiency of image processing during model ingestion, we extracted the pixel data from every DICOM file and converted it to PNG format. This extraction process was facilitated by MicroDICOM, a freely available software for viewing DICOM files.

**Figure 4:**
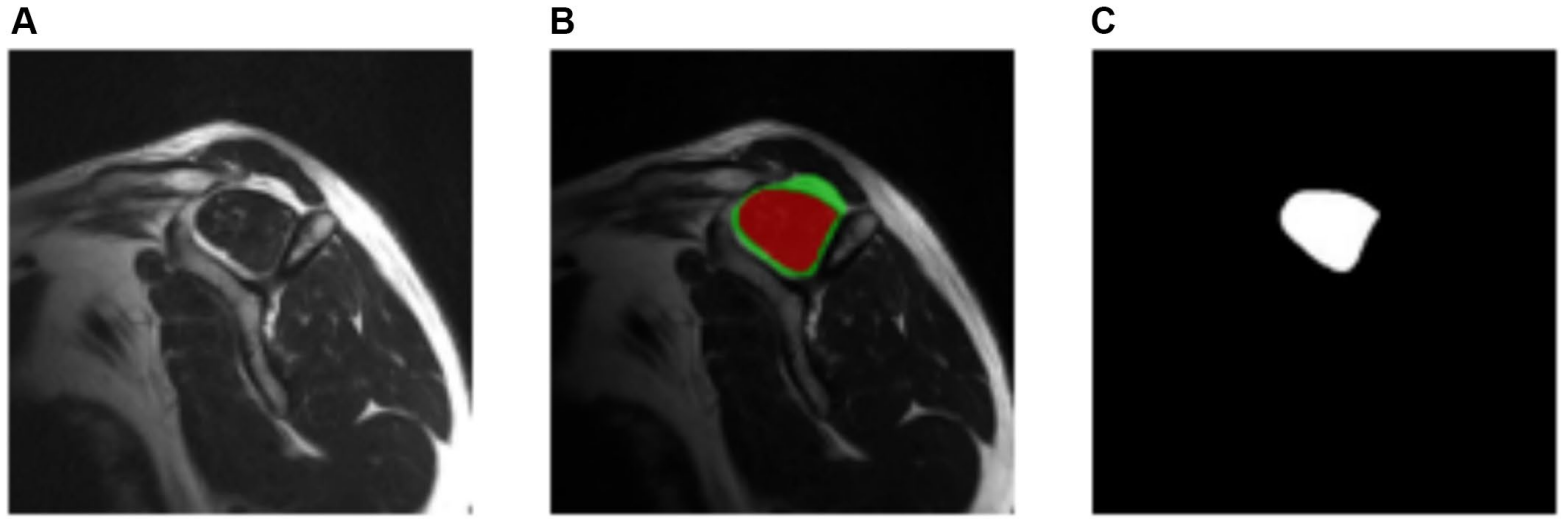
Manually segmentation process. **(A)** Original image. **(B)** Resulting segmentation masks. Supraspinous fossa in green, supraspinatus muscle in red. **(C)** Supraspinatus muscle mask.

The ITK-Snap3 software was utilized to generate the segmentation masks. In this case, separate masks were created for the supraspinous fossa area and the supraspinatus muscle area. Considering the specific evaluation of the fatty infiltration grade of the muscle based on the muscle area alone by physiologists, the focus was directed towards the supraspinatus muscle area mask for the subsequent steps. The final outcome of the segmentation process is visualized in Figure 4.

The data preparation process resulted in multiple images in PNG file format, each accompanied by its corresponding segmentation mask and label. Figure 1 provides a visual representation of the workflow involved in the data preparation.

#### Criteria for fatty infiltration

The criteria were based on Goutallier’s fatty infiltration definitions. According to the original paper, five levels of fatty infiltration were proposed, ranging from zero to four, to signify the qualitative presence of fat in the muscle. A level zero indicates the absence of fat in the muscle, while higher levels correspond to increasing fatty infiltration. Goutallier’s scale assigns higher values as the fatty infiltration intensifies. A level four indicates a higher amount of fat than muscle present.

As mentioned earlier, the objective is to assist clinicians in determining the risk associated with performing surgery based on the quality of the supraspinatus muscle. From a classifier perspective, this task can be viewed as a binary classification. In this study, Goutallier’s fatty infiltration levels zero or one were classified as “not risky,” while levels three or four were categorized as “risky.” Samples labeled as Goutallier level two were excluded from the analysis. This choice is based on previous research [see Saavedra et al. (20)] where it is shown that including Goutallier’s level 2 into a binary classification task does not significantly impact the performance of a classification model. Also, clinical relevance falls in correctly those cases where there is high or low level of fatty infiltration [see references (10) and (11)].

#### Proposed model

The proposed model is composed of two sequential neural network models that serve distinct purposes. Model A is designed to narrow down the region of interest in the MRI image by leveraging both the image and the segmentation mask as inputs. The U-Net model is proposed for this task (see next). Its primary objective is to predict the supraspinatus muscle area. The hypothesis is that this approach effectively eliminates irrelevant information from the image, thereby enhancing the performance of the second network. Following Model A (segmentation), Model B (classification task) takes the supraspinatus muscle area of the image as input and predicts the fatty infiltration level based on the Goutallier’s fatty infiltration level scale. An overview of the workflow is provided in Figure 2, while the subsequent subsections offer a detailed explanation.

Cross validation (*k*-fold) was performed during the training process. The total of 606 Y-view MRI shoulder images were grouped into five non overlapping folds. Each time, four folds were used as the training set and one as the validation set. Every fold was used four times as part of the training set and one time as part of the validation set. Fold composition was the same for both models (Model A and Model B). Model performance was computed as the average of those five training processes and 95% confidence intervals (CI) were obtained. In every training process the model with the lowest loss function value was considered the best model.

#### Model development and training

The proposed model was built using two sequenced architectures: U-Net (28) (Model A) and VGG-19 (29) (Model B). The first sub-model created the segmentation mask of the input image, and the second, performed the fatty infiltration classification for that same image. The selection of the VGG-19 model for the classification task is supported by previous research [see reference (20)] where it is shown that the VGG-19 is among the best CNN for fatty infiltration (among the tested models). Although the proposed framework follows sequential stages, the training process was performed in two steps. In the first step, we trained the segmentation model using every image and the corresponding segmentation mask as input.

The objective was for the model A to learn to predict the corresponding segmentation mask for an image that had not been seen previously. In the second step, a classification model was trained using the region of interest of the image and its corresponding label. Before feeding the classification model, automatic cropping of the image was performed, and only the region of interest was used as input for the classification model.

A repeated stratified *k*-fold cross-validation was performed in both steps. This method allowed us to use the entire dataset in the training process and minimize the influence of data selection, as occurs when using random train/validation/test splitting. The *k* value was set equal to 5 and, therefore, 5 non-intersecting groups were created at random. The proportion of every class in the original dataset was replicated in every group. Each time, four groups were used to create the training set and one was used to create the validation set.

The model performance was computed as the average of 5 training processes, and the corresponding confidence intervals were reported. Confidence intervals obtained from the cross-validation training process was used to assess robustness of the trained models. Due to the high imbalance of the dataset, the minority class was up sampled. In every training process, the smaller class was replicated until the proportion between classes was close to 1:1. The added images were copies of their originals but with slight differences in terms of rotation (±35°), horizontal flipping, and center cropping. The up-sampling process was carried out for the training data only. Figure 1 shows the workflow of the model training process.

Step 1: Training the segmentation model. For the segmentation task, a “U”-shaped neural network was built as described in Khouy et al. (28). The only difference is that (1, 1) padding was used in every convolutional layer to allow the network to utilize the entire image during the training process. The model was training for a maximum of 50 epochs and feeding the network with batches of five images at a time. We used binary cross-entropy loss, implemented in the PyTorch framework. The optimization algorithm used was Adam optimizer with its standard configuration. The learning rate was set to 10-5.

The segmentation process was performed using the U-Net model. The training hyperparameters were as follows: batch size = 8, maximum epochs = 50, input size = 224 × 224 (px), learning rate = 10^−3^, optimizer = Adam (standard configuration). The loss function used was the Dice loss, which was defined as:


(1) $$\mathrm{Dice}\;\mathrm{score}=2\times p\times t/\left(p^{2}+t^{2}\right)$$

(2) Diceloss=1−Dicescore

In Equation 1, “*p*” represents predicted values from the output, and “*t*” represents true values from the input. Basically, the Dice score (see Equation 2) measures the ratio of the intersection over the union for the resulting segmentation mask (30). The better the performance of the segmentation model, the higher the Dice score value. On the other hand, the Dice loss is the function to be minimized. The higher the value of the Dice score, the lower the value of the loss function.

Step 2. Training the classification model: The VGG-19 architecture was used for the classification task. We kept the convolutional layers of the model as the original and only the last layer of the fully connected layers was changed. Originally, the output of the VGG-19 architecture was 1,000 neurons. In our case we use only one output unit. That way, the model was able to perform the binary classification of the inputs.

To train the model, we used transfer learning. This means that all the weights of the original models trained on the ImageNet dataset were utilized. These weights were not optimized during the training process, and only the classifier layers were optimized. We employed the same maximum number of epochs, batch size, loss function, and optimizer as in the segmentation training process. A termination function was implemented to stop the training process if there was no improvement in the last 10 epochs. The best performance was saved and recorded. The only hyper-parameter that was optimized was the learning rate, and the best performance was achieved at 10^−5^. In the following section, we will present the output of both models, including the segmentation mask and a detailed explanation of the obtained metric values.

### Statistical analysis

Normality tests were conducted, and the analysis of statistical differences between groups utilized either the Mann–Whitney *U* test or *t*-test. A significance level of *p* < 0.05 was employed to establish statistical significance. Descriptive analysis of patient ages was performed, presenting the mean and standard deviation (m ± sd). Categorical data were expressed as percentages and frequencies.

The performance of the models was evaluated and compared based on accuracy, sensitivity, specificity, and area under the receiver operator curve (AU-ROC). A binary classifier produces either 0 or 1 for a given input, corresponding to the actual expected output. True positive (TP) was defined as the model correctly predicting the positive class. False positive (FP) refers to the model incorrectly predicting the positive class when it is actually negative. False negative (FN) occurs when the model incorrectly predicts the negative class when it is actually positive. True negative (TN) is when the model correctly predicts the negative class. Sensitivity, specificity and accuracy (Equations 3–5), were computed as follows:


(3) $$\mathrm{Sensitivity}\;\left(\mathrm{true}\;\mathrm{positive}\;\mathrm{rate}\right):TP/\left(TP+FN\right)$$

(4) $$\mathrm{Specificity}:TN/\left(TN+FP\right)$$

(5) $$\mathrm{Accuracy}:\left(TN+TP\right)/\left(TN+FP+FN+TP\right)$$

The AU-ROC measures the classifier’s performance regardless of the threshold used to convert probability scores into class decisions. The horizontal axis represents recall (sensitivity), while the vertical axis corresponds to precision, calculated as TP/(TP + FP). As both axes range from 0 to 1, the maximum value of the area under the curve inside the square is 1, indicating better classifier performance. A random classifier would have an AU-ROC equal to 0.5.

For metrics such as accuracy, sensitivity, specificity, and AU-ROC, 95% confidence intervals over the mean were calculated to assess model performance. All statistical analyses were conducted using the Python programming language.

## Results

### Sociodemographic characteristics

Male subjects presented 333 images, representing 55% of the sample. The patient’s average age was 55.1 ± 13.2 years. The data showed the presence of various types of Goutallier levels in MRI exams. An asymmetrical distribution of Goutallier grades was identified. A significant majority, exceeding 82% of the images, fell into grades 0 and 1, indicating a notable prevalence of low fatty infiltration: Goutallier 0 (66.50%), Goutallier 1 (18.81%), Goutallier 2 (8.42%), Goutallier 3 (3.96%), and Goutallier 4 (2.31%). Furthermore, the female group exhibited a higher frequency of samples in higher grades compared to the male group, although this disparity did not reach statistical significance. For more information, refer to Table 1.

#### Step 1. Segmentation

**Figure 5:**
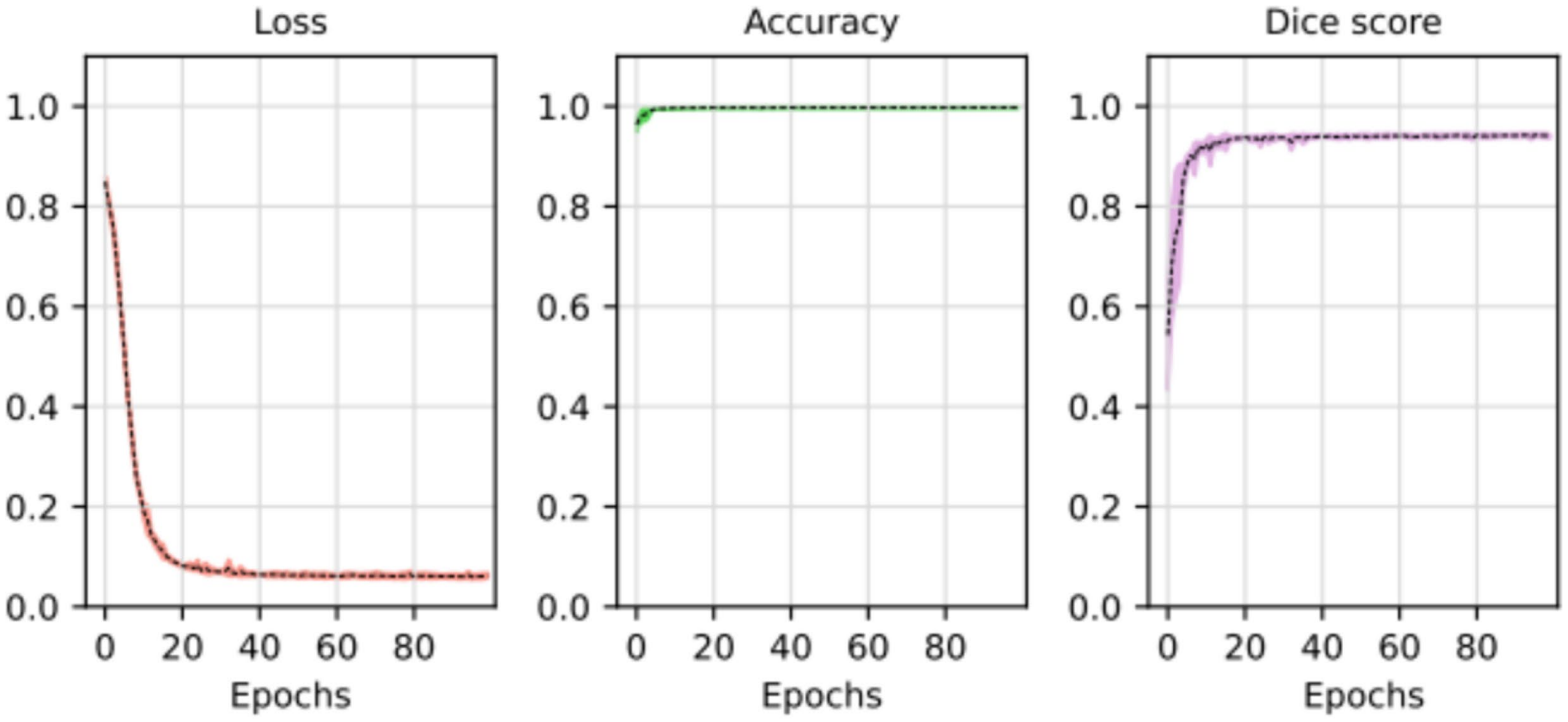
Loss, accuracy, and Dice score for the segmentation model. The average of the five training processes is shown in segment line. The color shadow shows the confidence interval (C.I. 95%).

At the outset of the training process, the loss value was recorded at 0.8498 ± 0.0102, serving as an initial baseline for assessing the model’s performance. As training progressed through successive epochs, a consistent reduction in the loss value was observed. Ultimately, post-training, the loss value significantly decreased to 0.0623 ± 0.0050. The training loss value (and other performance metrics) can be observed in Figure 5.

The substantial decline in the loss value reflects a considerable improvement in the model’s predictive accuracy. The reduction over the epochs suggests that the model became increasingly proficient at minimizing errors and refining its predictions. The tight standard deviations associated with the initial and final loss values underscore the reliability and consistency of the observed improvements.

These results imply that the deep learning model underwent effective training, optimizing its ability to generalize patterns and make accurate segmentation tasks. The detailed evolution of the loss value throughout the epochs provides a quantitative measure of the model’s learning process and its enhanced performance at the training’s conclusion.

**Figure 6:**
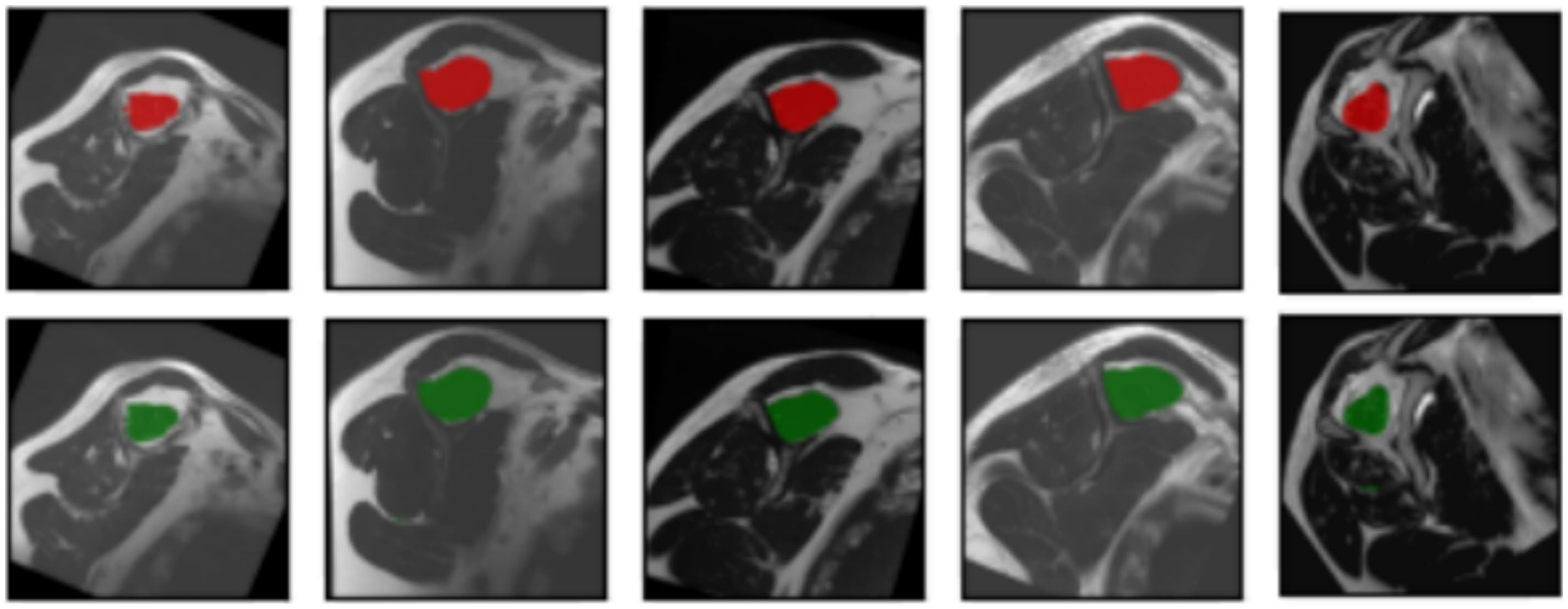
Input masks and the respectively, output masks obtained from the U-Net model. The original masks are shown in red; the resulting masks are shown in green. For each input image showed in every column of the first or the third row, the corresponding output mask from the U-Net is showed on the same column in the second and fourth rows, respectively.

The segmentation task performed by the model can be observed in Figure 6. The original input mask is highlighted in red, and the model’s output mask is highlighted in green. The background of each case displays the original image. Before making modifications, the images were rotated before being fed into the segmentation model. This rotation aims to prevent the model from memorizing specific patterns and, instead, encourages it to learn more generalized concepts from the data.

In most cases, the resulting segmentation mask (in green) closely resembles the original input segmentation mask (in red). This suggests that the model effectively learned to perform the segmentation task without memorizing specific samples from the training dataset. The similarity between the masks indicates that the model has generalized correctly and can apply its knowledge to new images effectively. In this sense, the model efficiently minimized errors during the training process, as indicated by the computed average loss value of 0.0587. This low loss value is crucial because it signifies the model’s ability to consistently converge toward accurate predictions. The small standard deviation of 0.0048 further emphasizes the precision and stability of the model’s training, reinforcing its reliability in capturing intricate patterns within the data. At the same time, the model shows its proficiency in correctly classifying instances with an average accuracy of 0.9977. With a minimal standard deviation of 0.003, the model also shows consistent accuracy across various data points. These findings highlight the robustness of the model in performing precise segmentation tasks. Finally, the model achieved an average Dice score of 0.9441, indicative of its efficacy in capturing the spatial agreement between predicted and ground truth segmentations. A small standard deviation of 0.0035 shows the model’s stability in consistently achieving high Dice scores. These results affirm the model’s performance in image segmentation tasks. For more details, please refer to Table 2.

**Table 2:** Segmentation results.

	Loss	Accuracy	Dice score
Average	0.0587	0.9977	0.9441
S.D	0.0048	0.003	0.0035
CI. (95%)	0.0586 ± 0.0042	0.9977 ± 0.0002	0.9441 ± 0.0031

Loss, accuracy, and Dice score were computed as the average of five training processes. Confidence interval calculated at *α* = 0.05.

#### Step 2. Classification

**Figure 7:**
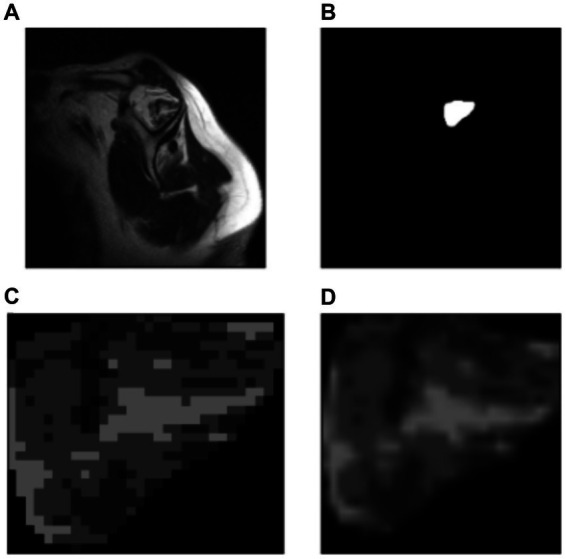
Automatic cropping process. **(A)** Original image. **(B)** Output mask from the U-Net model. **(C)** Cropped region of interest from the original image (ROI). **(D)** Resized region of interest (224 × 224 px).

Figure 7 shows the original image (A) and the segmentation mask obtained from the U-Net model (B). Then using that segmentation mask, the region of interest was cropped (automated process) from the original image (C). Finally, a resizing function was applied to the image, resulting in (D). This pre-processing allowed the model to decide considering only the supraspinatus muscle, similarly as how the clinicians do.

**Figure 8:**
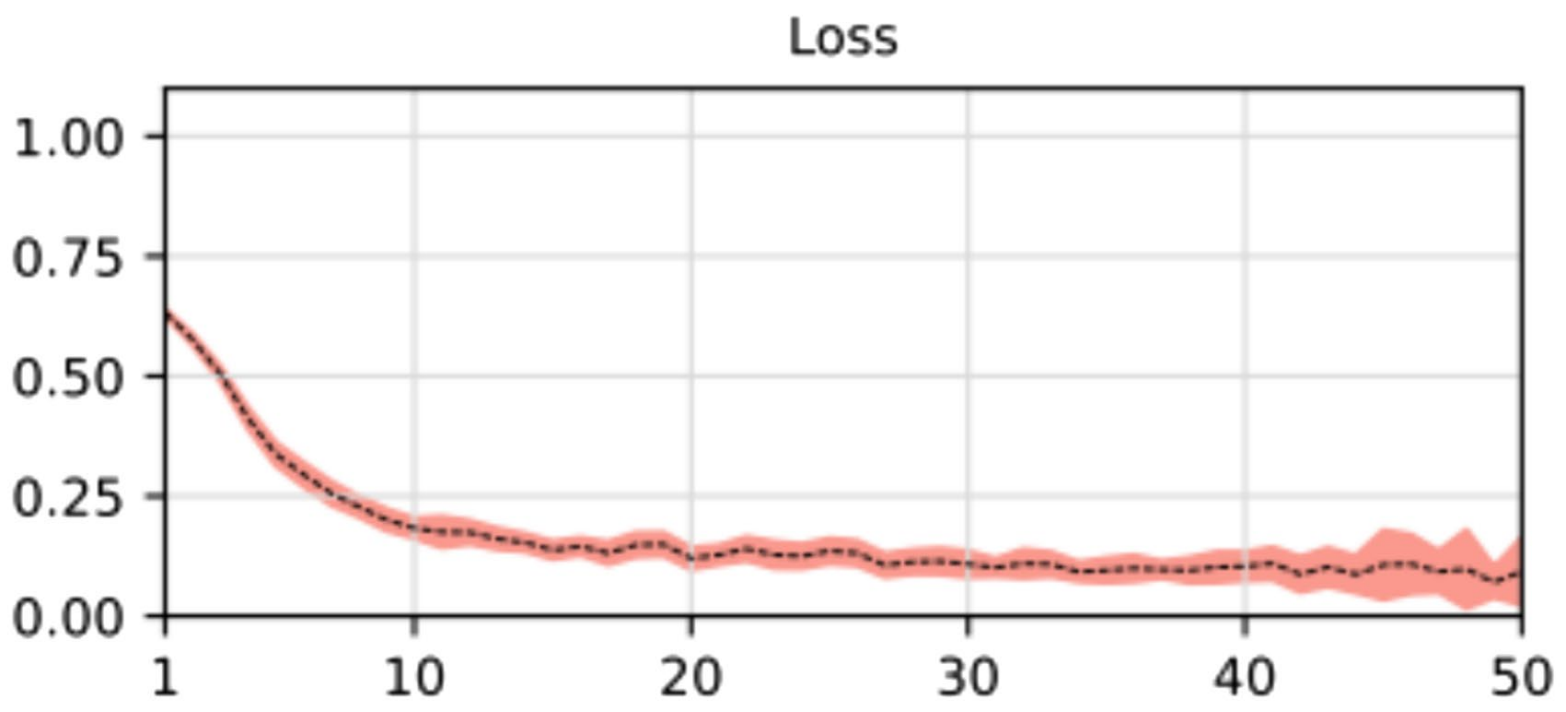
Average and confidence interval (*α* = 0.05) for the classification validation loss over five-folds cross-validation training processes. Average is shown in segmented line, and confidence interval is shown in shadow.

**Figure 9:**
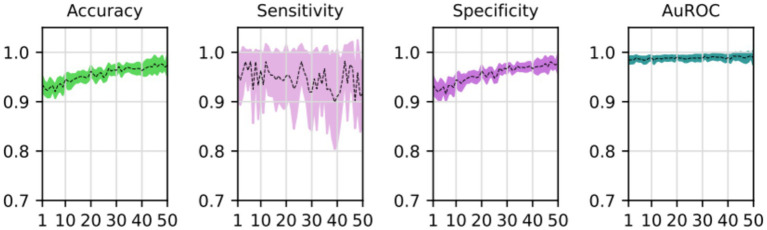
Average and confidence interval (*α* = 0.05) for the classification validation accuracy, sensitivity, specificity, and area under the ROC curve (AUROC), over five-folds cross-validation training processes. Average is shown in segmented line, and confidence interval is shown in shadow.

During the training process, the loss function value for the validation set was monitored. At the beginning of the training process the loss value was 0.6645 ± 0.0228, decreasing to 0.01178 ± 0.0037 after the training process was concluded. The accuracy, sensitivity, specificity and AUROC were computed as the average of the model performance over the validation set in each of the five training processes of the *k*-fold. Table 3 shows the results for those metrics in terms of the confidence interval (*α* = 0.05). As shown, every metric value is above 0.9 (on average), hence showing a good binary classification performance of fatty infiltration of the supraspinatus muscle based on Goutallier’s fatty infiltration scale. In particular, the accuracy reached a level of 97.3% with a 0.023 95% CI, showing high precision (low variability). Even though the results show a higher value of specificity compared to sensitivity, the difference could increase if no oversampling (or other data-balancing technique) was used. In this case, sensitivity reached a level of 90% with 0.98 95% CI, while the sensitivity showed a high level of 97.9% with a low 95% CI of 0.02. Finally, the balancing of these two metrics was computed by the AU-ROC, which has an average level of 99% with a low 95% CI of 0.009, indicating a high level of capability to differentiate risky from non-risky levels of fatty infiltration based on automated segmented images from the U-Net model (see Figures 8, 9).

**Table 3:** Classification model results.

	Loss	Accuracy	Sensitivity	Specificity	AUROC
Average	0.1065	0.9731	0.9000	0.9788	0.9903
S.D	0.0584	0.0263	0.1118	0.0293	0.0105
CI. (95%)	0.1065 ± 0.0512	0.9731 ± 0.0230	0.9000 ± 0.0980	0.9788 ± 0.0257	0.9903 ± 0.0092

Confidence interval computed from the validation set of the five training processes at *α* = 0.05. The loss, accuracy, sensitivity, specificity, and area under the ROC curve (AUROC) are shown.

Results of the proposed automated two steps training model shows that the segmentation model could first learn how to find the region of interest (supraspinatus muscle). Then, the classification model could learn how to classify the input, based on that region of interest, as risky or not risky. Cropping the region of interest before feeding the classifier, allowed the model to learn as clinicians do. However, the two step process proposed here shows a small reduction in classification performance (sensitivity, specificity, accuracy and AU-ROC) when compared to different CNN trained on the same data but considering manual segmentation of the ROI [see Saavedra et al. (20) for details]. Table 4 shows the comparison of the two step proposed model (U-NET + VGG-19) with VGG-19, ResNET-50 and Inception-v3 models. As noted, given that manual segmentation done by professional clinicians and medical expert is more accurate that segmentation performed by U-NET, errors from the U-NET model are passed on to the VGG-19 classification model, resulting a slightly lower performance. However, the (almost insignificant) reduction of performance is valid as the proposed model completely automates the process of identifying the level of fatty infiltration, reducing hence the need for lengthily process of manual segmentation of the ROI of the supraspinatus muscle.

**Table 4:** Classification model comparison with literature.

	Loss	Accuracy	Sensitivity	Specificity	AUROC
Proposed model	0.106 ± 0.051	0.973 ± 0.023	0.900 ± 0.098	0.978 ± 0.025	0.990 ± 0.009
VGG-19	0.096 ± 0.010	0.973 ± 0.006	0.947 ± 0.039	0.975 ± 0.006	0.991 ± 0.003
ResNet-50	0.123 ± 0.011	0.976 ± 0.006	0.925 ± 0.053	0.980 ± 0.006	0.992 ± 0.003
Inception-v3	0.102 ± 0.009	0.974 ± 0.007	0.869 ± 0.085	0.981 ± 0.006	0.991 ± 0.004

Confidence interval computed from the validation set of the five training processes at *α* = 0.05. The loss, accuracy, sensitivity, specificity, and area under the ROC curve (AUROC) are shown. Models used for comparison are obtained from Saavedra et al. (20).

## Discussion

This article introduces a novel deep-learning framework for assessing the degree of fatty infiltration in the supraspinatus muscle. The framework performs two main tasks: segmenting the region of interest and classifying the level of fatty infiltration on a five-level scale proposed by Goutallier et al. (10) based on the automated segmentation process. To achieve this, we developed two sub-models: the first based on the U-Net architecture for segmentation, and the second based on the VGG-19 architecture with modified classifier layers for binary classification. We first trained the segmentation sub-model using segmentation masks and then trained the classification sub-model using the labels associated with the fatty infiltration diagnosis. We used transfer-learning weights to train both sub-models. The binary output of the model (0 or 1) was interpreted as “not risky” or “risky,” respectively, with higher levels of fatty infiltration indicating a greater risk of re-tear or poor surgical outcomes.

Our model achieved strong performance thanks to the implementation of transfer learning and k-fold cross-validation techniques. By leveraging these approaches, we were able to reduce the number of parameters requiring optimization and utilize the full dataset for both training and validation purposes, effectively guarding against overfitting issues given our relatively small dataset of slightly more than 600 samples. However, some research has made efforts to optimize the process of hyperparameter optimization (31). Still, it’s worth noting that relying on transfer learning from a pre-trained model on the ImageNet dataset may not always represent the most ideal solution. This can be seen as a possible limitation of the relatively small sample of images obtained for this study. Future research should focus on evaluating the effect of the proposed training process. This is needed to understand if the high accuracy levels obtained in this research are driven by transfer learning and data augmentation techniques or to identify if the task or segmenting and classifying fatty infiltration in the supraspinatus muscle is a simpler task compared to more complex images (such as X-rays or ultrasounds of different body or biological structures).

In the medical domain, obtaining labeled data or segmentation masks for images can be challenging. Meanwhile, radiological reports are abundant and readily available. Manual labeling or segmentation is a labor-intensive process, but leveraging the valuable information contained in reports can facilitate model training without significant human effort. Another approach worth considering is unsupervised learning, which can enable the model to learn without relying on fully labeled or segmented data. Additionally, using transfer learning with a pre-trained model in a related domain, such as shoulder MRI images or MRI images more broadly, has the potential to enhance the model’s performance.

Deep learning models have been increasingly applied in radiology, with the U-Net (28) being a particularly popular choice for segmentation tasks. One example of this is Taghizadeh et al. (27), who employed the U-Net model to assess muscle degeneration levels in CT scans. Through a supervised training approach with annotated data, they successfully segmented the structures and characterized the pre-morbid state based on clinical information. By comparing these two states, they were able to quantify the degree of muscle degeneration.

Medina et al. (22) proposed two sequential models trained in a supervised manner via transfer learning from a model pre-trained on the ImageNet dataset. Both models had all their weights initially frozen except for the classifier layers, which were optimized by training the network on a shoulder MRI dataset. Model A aimed to identify the best image in a series depicting the rotator cuff muscles, while Model B focused on segmenting the four rotator cuff muscles. Model A was constructed using the Inception-v3 architecture, while Model B was based on the VGG19 architecture.

Kim et al. (26) proposed a unique approach for assessing muscle atrophy in the supraspinous fossa by measuring the occupation ratio (O.R.) of the supraspinatus muscle. They used a VGG19-like network to segment the region of interest with annotated data, but gaps in the muscle area obtained from the model required filling with a post-processing algorithm. The authors then determined the stage of muscle atrophy based on the O.R. (stage I: O.R. ≥ 0.6; stage 2: 0.4 ≤ O.R. ≤ 0.6; stage 3: O.R. > 0.4). Although this method did not assess the fatty infiltration grade precisely, it was still a valuable contribution.

Ro et al. (23) also utilized the VGG19 model to perform a segmentation task for identifying the region of interest. To convert the grayscale image into a binary representation, they applied Otsu’s thresholding (32), a technique commonly used to separate the foreground (fat) from the background (muscle) in the image. However, as in other studies, post-processing was required, and the results were not directly applicable to a fatty infiltration scale like Goutallier’s.

This study has some limitations that must be considered. Firstly, a domain bias might have been introduced to the prediction because the MRI images and natural images used in the training process came from very different dataset. While we used the cross-validation technique to overcome the over-fitting problem, we were unable to test our data on an external dataset, which could limit the model’s generalizability if it is intended to be used in a production environment. To address this issue, future studies could focus on training the model on a larger set of MRI images to improve both the model’s performance and the clinician’s reliance on an artificial intelligence-driven solution. Also, it is important to consider that in order to bring these new models and technologies to production environment (deployment), computational resources must be considered as the models must be retrained as new data comes in. This also helps improving and refining the deployed models. To properly do this, deployment environments (hospitals or clinics) must be equipped with appropriate computational tools (servers or computers) to efficiently manage the update of models, which also increase in complexity and computational resources needed as more data becomes available. Additionally, the manual labeling task was performed by only one trained radiologist, which might limit the reliability of the ground truth. To improve the accuracy and consistency of the labeling process, future studies could consider involving multiple trained radiologists in the task and comparing the model’s performance with that of the professionals. Finally, further efforts should be pursued to evaluate the feedback-loops during the training process of the proposed two-stage algorithm. This research did not focus on the possible improvements of the segmentation and classification models when feeding their results and predictive errors, similar to what boosting or sequential machine learning algorithms do.

In summary, this study analyzed a dataset of MRI images to assess fatty infiltration levels in the supraspinatus muscle among patients with rotator cuff conditions. We proposed a two-step training method using deep learning models, which demonstrated strong performance in segmentation and classification tasks. These findings indicate the potential of these models for accurate and reliable evaluation of musculoskeletal conditions in similar clinical settings.

## Data Availability

The data analyzed in this study is subject to the following licenses/restrictions: data might be requested to authors and it will be sent if authorized by corresponding authorities as they are images of patients. Requests to access these datasets should be directed to guillermo.droppelmann@meds.cl.
